# Skeletal Muscle Ribosome and Mitochondrial Biogenesis in Response to Different Exercise Training Modalities

**DOI:** 10.3389/fphys.2021.725866

**Published:** 2021-09-10

**Authors:** Paulo H. C. Mesquita, Christopher G. Vann, Stuart M. Phillips, James McKendry, Kaelin C. Young, Andreas N. Kavazis, Michael D. Roberts

**Affiliations:** ^1^School of Kinesiology, Auburn University, Auburn, AL, United States; ^2^Department of Kinesiology, McMaster University, Hamilton, ON, Canada; ^3^Department of Cell Biology and Physiology, Edward Via College of Osteopathic Medicine, Auburn, AL, United States

**Keywords:** skeletal muscle, ribosomes, mitochondria, AMP-activated protein kinase, mechanistic target of rapamycin, exercise training, concurrent training

## Abstract

Skeletal muscle adaptations to resistance and endurance training include increased ribosome and mitochondrial biogenesis, respectively. Such adaptations are believed to contribute to the notable increases in hypertrophy and aerobic capacity observed with each exercise mode. Data from multiple studies suggest the existence of a competition between ribosome and mitochondrial biogenesis, in which the first adaptation is prioritized with resistance training while the latter is prioritized with endurance training. In addition, reports have shown an interference effect when both exercise modes are performed concurrently. This prioritization/interference may be due to the interplay between the 5’ AMP-activated protein kinase (AMPK) and mechanistic target of rapamycin complex 1 (mTORC1) signaling cascades and/or the high skeletal muscle energy requirements for the synthesis and maintenance of cellular organelles. Negative associations between ribosomal DNA and mitochondrial DNA copy number in human blood cells also provide evidence of potential competition in skeletal muscle. However, several lines of evidence suggest that ribosome and mitochondrial biogenesis can occur simultaneously in response to different types of exercise and that the AMPK-mTORC1 interaction is more complex than initially thought. The purpose of this review is to provide in-depth discussions of these topics. We discuss whether a *curious competition* between mitochondrial and ribosome biogenesis exists and show the available evidence both in favor and against it. Finally, we provide future research avenues in this area of exercise physiology.

## Introduction

Research interest in the fields of ribosome and mitochondrial biogenesis has been growing considerably over the last decades. While the number of overall publications listed on MEDLINE has been increasing steadily during the last 20years (~200% increase when comparing 2020–2000), during the same period, there was an even greater increase (over 2,500%) in the number of publications with the search terms “ribosome biogenesis” or “mitochondrial biogenesis.” Much of the research in “ribosome biogenesis” and/or “mitochondrial biogenesis” has dealt with cancer biology (Derenzini et al., 2017; Vanderveen et al., 2017; Pelletier et al., 2018), aging (Tiku and Antebi, 2018; Correll et al., 2019; Roque et al., 2020), and other disciplines unrelated to exercise physiology. However, in recent years, several exercise physiology laboratories have been utilizing more mechanistic molecular tools to study the adaptations that occur with exercise to discern the well documented health and/or performance benefits following exercise.

Ribosome and mitochondrial biogenesis are both complex processes. A detailed description of the molecular underpinnings of each process is beyond the scope of this review and readers are referred to other excellent reviews on the topics [ribosome biogenesis (Henras et al., 2015; Kressler et al., 2017), mitochondrial biogenesis (Jornayvaz and Shulman, 2010; Bouchez and Devin, 2019)]. For the purpose of this review, ribosome biogenesis refers to the *de novo* synthesis of ribosomes, a process that involves the transcription and processing of rRNA and the assembly of several ribosomal proteins. The rate-limiting step of ribosome biogenesis is thought to be generation of the 45S pre-rRNA by RNA Polymerase I (Kopp et al., 2007). This precursor is then processed, yielding the 18S, 5.8S, and 28S mature rRNA transcripts. These transcripts are exported to the nucleus and associate with 5S rRNA and different ribosomal proteins resulting in the assembly of the mature ribosome (Kressler et al., 2017). Mitochondrial biogenesis, is accomplished through the recruitment of newly synthesized mitochondrial proteins to existing organelles, which can grow and divide (Ryan and Hoogenraad, 2007; Miller and Hamilton, 2012). Mitochondrial biogenesis involves the transcription of proteins encoded by both nuclear and mitochondrial genomes. Considered a major regulator of mitochondrial biogenesis, peroxisome proliferator-activated receptor-γ coactivator 1α (PGC-1α) activates nuclear respiratory factors, increasing nuclear transcription of mitochondrial genes (Ryan and Hoogenraad, 2007). These nuclear respiratory factors activate mitochondrial transcription factor A (TFAM), which promotes the transcription and replication of mitochondrial DNA (Wu et al., 1999).

Importantly, researchers frequently use activation markers of cell signaling pathways, mRNA expression, protein levels, and/or enzymatic activity as a measure of mitochondrial biogenesis. However, as nicely discussed by Miller and Hamilton (2012), such variables present important limitations (i.e., measure of organelle content instead of biogenesis process *per se*, disregarding degradation processes), and although informative, are not direct measures of biogenesis. The only direct measure of mitochondrial biogenesis currently available is the measure of mitochondrial protein synthesis using stable isotopic tracers. Similarly, ribosome biogenesis is usually assessed through measurements of cell signaling, mRNA expression, protein levels, total RNA and/or rRNA levels, variables that are at best only indirect measures of biogenesis. Tracer methodologies have also been developed and used to measure *de novo* ribosomal biogenesis (Brook et al., 2017). However, considering the fact that only few exercise training studies have used direct measures of ribosome and/or mitochondrial biogenesis, studies using indirect measures will be included and discussed in the current review. Readers are strongly encouraged to consider whether direct or indirect measures were used when interpreting the results of the studies presented herein.

Resistance and endurance training increase skeletal muscle ribosome biogenesis and mitochondrial biogenesis, respectively. Mitochondrial biogenesis increases aerobic capacity (Costill et al., 1976; Burgomaster et al., 2008; Yeo et al., 2008; Murias et al., 2011; Cochran et al., 2014; Vigelso et al., 2014), and ribosome biogenesis has been associated with skeletal muscle hypertrophy [reviewed in (Chaillou et al., 2014; Wen et al., 2016; Mcglory et al., 2017; Bamman et al., 2018; Roberts et al., 2018a; Figueiredo and Mccarthy, 2019; Kim et al., 2019)]. It is generally believed that skeletal muscle adaptations to exercise are highly specific. Increased ribosome biogenesis with resistance training is seemingly prioritized over mitochondrial biogenesis (Wilkinson et al., 2008; Figueiredo et al., 2021), while there is evidence to suggest increased mitochondrial biogenesis with endurance training is prioritized over ribosome biogenesis (Morrison et al., 1989; Gibala et al., 2009). In addition, an interference effect may occur if both endurance and resistance exercise are included in the same training session or program (i.e., concurrent training). For example, published reports show that endurance training compromises muscle hypertrophy response to resistance training (Kraemer et al., 1995; Jones et al., 2013) and, although researchers have tried to unveil the mechanisms underlying the interplay between resistance and endurance training, the molecular underpinnings of these observations are still unclear. However, evidence suggests that both processes can occur simultaneously (Tang et al., 2006; Fyfe et al., 2016, 2018). Therefore, the purpose of this review is to discuss whether this *curious competition* between mitochondrial and ribosome biogenesis exists during different exercise training programs (i.e., only resistance training, only endurance training, or concurrent training) and show the available evidence both in favor and against it. We also discuss whether both processes can concomitantly increase with certain exercise training paradigms and provide future research avenues in this area of exercise physiology.

## Ampk and Mtor Signaling Help Regulate Mitochondrial and Ribosome Biogenesis, Respectively

Two critical signaling proteins that facilitate the adaptive responses to exercise training include the 5' AMP-activated protein kinase (AMPK) and the mechanistic target of rapamycin (mTOR). As an important regulator of cellular energy homeostasis, AMPK is a hetero-trimeric cytosolic enzyme with a catalytic α-subunit and regulatory β and γ subunits. The α-subunit phosphorylates cytoplasmic and nuclear proteins to affect the expression of various mRNAs. High adenosine monophosphate (AMP) concentrations during exercise (as a result of high ATP turnover) lead to increased binding of AMP with AMPK (Richter and Ruderman, 2009), but it has been shown that ADP could also activate AMPK (Oakhill et al., 2011). In addition, glycogen interacts with the β-subunit of AMPK, and muscle glycogen depletion during exercise results in the loss of the interaction between these molecules, which increases AMPK activity (Steinberg et al., 2006). Stress-responsive proteins, such as serine/threonine kinase 11 and calcium/calmodulin-dependent protein kinase 2, can also act to phosphorylate AMPK at the Thr^172^ residue and increase its activity (Richter and Ruderman, 2009). Evidence in multiple cell lines and tissues suggests that increased AMPK signaling facilitates mitochondrial gene expression to provide for mitochondrial biogenesis (Reznick et al., 2007; Yan et al., 2013; Marin et al., 2017). In this regard, endurance exercise studies with rodents and humans have shown AMPK signaling and mRNAs involved in mitochondrial biogenesis increase hours following exercise (Fujii et al., 2000; Atherton et al., 2005; Jorgensen et al., 2005). Additionally, researchers have used the muscle-specific double knockout AMPK β1 and β2 mouse model (β1β2M-KO) to demonstrate functional AMPK is critical in maintaining muscle mitochondrial content (O'neill et al., 2011).

The mechanistic target of rapamycin complex 1 (mTORC1) signaling pathway is widely recognized as a regulatory hub for overload-induced skeletal muscle hypertrophy (Goodman, 2019). mTORC1 is a multi-subunit complex that consists of the mTOR protein as well as Raptor and mTOR associated protein LST8 homolog (mLST8; Saxton and Sabatini, 2017). Like AMPK, active mTORC1 complexes possess kinase activity to phosphorylate downstream proteins that facilitate the assembly and initiation of translation-competent ribosomes. Bodine et al. (2001) were the first to demonstrate mTOR signaling was required for muscle hypertrophy. Specifically, the authors administered rapamycin (an mTOR inhibitor) to mice, and observed synergist ablation-induced hypertrophy was completely abrogated in the plantaris muscle. Human studies have since shown that phosphorylation of mTOR and its downstream substrates (i.e., p70s6k, 4EBP1) are critically involved in facilitating post-exercise increases in muscle protein synthesis (Drummond et al., 2009; Gundermann et al., 2014). Further, acute increases in mTOR signaling markers following one bout of resistance exercise are associated with muscle hypertrophy following weeks of resistance training (Terzis et al., 2008; Hulmi et al., 2009; Mayhew et al., 2009; Mitchell et al., 2013). Aside from upregulating muscle protein synthesis, more recent evidence suggests mTOR signaling regulates ribosome biogenesis across multiple cell lines [reviewed in (Mayer and Grummt, 2006)]. Notably, Nader et al. (2005) were the first to demonstrate this mechanism occurs in skeletal muscle cells *in vitro*. Von Walden et al. (2016) later demonstrated that mTOR signaling enhances ribosome biogenesis in skeletal muscle cells *in vitro* by modifying chromatin at the rDNA promoter. For further information on this topic, readers are encouraged to refer to other excellent reviews (Kim et al., 2019; Von Walden, 2019).

## What Evidence is There Suggesting Mitochondrial and Ribosome Biogenesis May Compete?

Several lines of evidence exist suggesting skeletal muscle mitochondrial and ribosome biogenesis *may* compete at the molecular level in response to different modes of exercise training. For instance, we have reported that Otsuka Long-Evans Tokushima Fatty rats exposed to 12weeks of treadmill training demonstrated ~60% lower total RNA per mg wet tissue (a surrogate of skeletal muscle ribosome density) compared to untrained animals (Romero et al., 2017), and data from these same animals showed skeletal muscle citrate synthase activity (a surrogate of mitochondrial volume) was ~16% higher in trained vs. untrained animals (Martin et al., 2012). While we did not assess markers of AMPK activation it is notable that others have shown treadmill running results in acute increases in markers of AMPK activity following exercise (Ruderman et al., 2003). Other rodent studies partially agree with our findings. For instance, Morrison et al. (1989) reported that rats that underwent 2weeks of treadmill training had greater hindlimb citrate synthase activity (~40%, *p*<0.05) compared to untrained rats, while 18S rRNA (a surrogate of ribosome density) was similar between groups. Hayase and Yokogoshi (1992) reported that rats that underwent 7days of treadmill exercise had non-significantly lower levels of total RNA/mg protein in the mixed gastrocnemius muscle (−5.6%, *p*=0.060) and soleus muscle (−4.7%, *p*=0.111) compared to untrained rats.

Regarding human studies, transcriptomic results from the 20-week HERITAGE cardiovascular training study indicated that certain ribosomal mRNAs in the vastus lateralis were downregulated from pre-to post-training (Teran-Garcia et al., 2005). Additionally, Wilkinson et al. (2008) used a 10-week unilateral leg training protocol to demonstrate the differential molecular adaptations to resistance vs. endurance training. Specifically, 10 healthy men with minimal training > 8months prior to the initiation of the study trained one leg using the knee extensor exercise (2–3days per week) and the other leg using a cycle ergometer (2–3days per week). The authors reported that basal myofibrillar protein synthesis rates increased from pre- to post-intervention within the resistance-trained leg only (~0.08%/h at POST vs. ~0.06%/h at PRE). Myofibrillar protein synthesis rates were also greater at the 10-week time point in the resistance vs. endurance-trained leg. While markers of ribosome biogenesis were not assessed, these data suggest resistance training may have increased ribosome density *via* biogenesis given the ~30% increase in basal myofibrillar protein synthesis rates. These data additionally suggest ribosome biogenesis was likely unaffected with endurance training. A recent study conducted by Figueiredo et al., (2021) supports the competition between mitochondrial and ribosome biogenesis theory. The authors investigated the genetic and epigenetic regulation of ribosome biogenesis with either endurance or resistance exercise and found that markers of ribosome biogenesis were increased with resistance exercise but decreased with endurance exercise (30min post-exercise). In addition, the authors reported that, in general, resistance exercise activated the mTOR pathway while endurance exercise activated the AMPK pathway. Collectively, these studies suggest endurance training does not alter ribosome biogenesis or may interfere with certain aspects of the process. However, more human endurance training studies are needed before definitive conclusions can be drawn.

Despite sparse evidence linking endurance training to unaltered or decreased ribosome biogenesis, several human studies have shown that resistance training increases ribosome density (as measured by total RNA per mg tissue; Kadi et al., 2004; Figueiredo et al., 2015; Stec et al., 2016; Brook et al., 2017; Reidy et al., 2017; Mobley et al., 2018; Hammarstrom et al., 2020). Separate reports have also shown that resistance training does not alter or decreases mitochondrial volume (as measured by citrate synthase activity assays or transmission electron microscopy; Macdougall et al., 1982; Luthi et al., 1986; Tesch et al., 1987; Parise et al., 2005; Porter et al., 2015). It is uncommon for the same study to report both variables. However, two human studies from our laboratory have examined changes in markers of skeletal muscle ribosome density and mitochondrial volume in response to resistance training. In one study, untrained young men participated in 12weeks (3days per week) of full-body resistance training (Roberts et al., 2018b), and following training, total RNA per mg tissue (vastus lateralis) increased by 23% (*p*<0.05), while vastus lateralis citrate synthase activity non-significantly decreased by 11% (*p*=0.064). Similar to these findings, we reported 6weeks of unaccustomed high volume resistance training in previously-trained young men increased vastus lateralis total RNA per mg tissue by 28% (*p*<0.05). In contrast, vastus lateralis citrate synthase activity decreased by 12% (*p*<0.05; Haun et al., 2019). Critically, both studies suggest ribosome biogenesis occurred with unaccustomed resistance training, whereas mitochondrial biogenesis either did not occur or was delayed relative to increases in myofiber hypertrophy. In addition, Hanson et al. (2019) found that performing a bout of endurance exercise before resistance exercise led to an acute decrease in markers of ribosome biogenesis compared to resistance exercise alone. However, it is important to note that markers of ribosome biogenesis were restored 3h post-exercise.

To summarize, several human studies suggest that unaccustomed resistance training increases ribosome density (likely through increased ribosome biogenesis), whereas mitochondrial density remains constant or decreases. Whether or not decrements in citrate synthase activity in these studies resulted from “mitochondrial dilution” *via* skeletal muscle hypertrophy rather than a decrease in mitochondrial biogenesis and/or a loss in mitochondria is debatable and is discussed elsewhere (Groennebaek and Vissing, 2017). Notably, most studies only used measures of ribosome and/or mitochondrial content or other indirect measures of biogenesis and their results should be interpreted with caution. Given the overall lack of data in this area, more research is needed to interpret the relevance of these findings.

## Why Would Mitochondrial and Ribosome Biogenesis Compete with One Another in Response to Exercise Training?

Ample molecular evidence exists to explain why mitochondrial and ribosome biogenesis may compete with one another during periods of exercise training. First, AMPK mechanistically blocks mTORC1 signaling through direct phosphorylation of the complex (Shaw, 2009) as well as through the phosphorylation and activation of the hamartin-tuberin (TSC1/2) complex [reviewed in (Shaw, 2009)], which is an upstream inhibitor of mTORC1 signaling. Given the proposed role mTORC1 signaling has on skeletal muscle ribosome biogenesis, it seems plausible that this process is impaired during situations of heightened AMPK signaling. In support of this hypothesis, we have reported that treating C_2_C_12_-derived myotubes with 5-aminoimidazole-4-carboxamide ribonucleotide (AICAR, a stimulator of AMPK activity) for 6hours reduced 47S pre-rRNA levels by 16% compared to vehicle-treated cells (Mobley et al., 2016); notably, while a one-way ANOVA with multiple cell culture treatments indicated no difference between the groups in our publication, a direct comparison between AICAR and vehicle-treated cells indicated *p*<0.05 between these two conditions. Researchers have also reported similar phenomena in other cell lines. For instance, several AMPK activators (e.g., phenformin, resveratrol, and AICAR) have been shown to disrupt nucleolar organization and inhibit ribosomal RNA synthesis in LLC-PK1 kidney proximal tubule epithelial cells (Kodiha et al., 2014). In HEK293T cells, glucose deprivation-induced AMPK activation has been reported to lead to increased phosphorylation of the RNA polymerase I-associated transcription factor TIF-IA at Ser^635^ (Hoppe et al., 2009). This phosphorylation event reduced the interaction of TIF-IA with other transcription factors and ultimately reduced the assembly of functional transcription initiation complexes at the rDNA promoter. Others have also shown a reduction in ribosome biogenesis in COS7 and HEK293 cells and transgenic mice overexpressing γ2-AMPK (Cao et al., 2017). Thus, it is apparent that a conserved outcome of AMPK activation in several cell types involves inhibition of ribosome biogenesis.

Evidence also exists suggesting mTORC1 signaling may reduce certain aspects of mitochondrial biogenesis. For instance, (Deepa et al., 2013) reported mRNAs involved with mitochondrial biogenesis (i.e., *Ppargc1a*, *Nrf1*, and *Esrra*) increased in the white adipose tissue of female *db/db* mice administered rapamycin (an mTOR inhibitor) for 6months. Chiao et al. (2016) reported that cardiac muscle mitochondrial biogenesis markers (i.e., PPARGC1A and TFAM protein levels) increased during the first 2weeks of a 10-week rapamycin feeding experiment in mice. There are also data showing mTORC1 signaling may disrupt autophagy, which in turn may affect mitochondrial remodeling (Choi et al., 2012). This is relevant to the competition paradigm given that autophagy is critical for mitochondrial remodeling and function in skeletal muscle cells *in vitro* (Sin et al., 2016). Furthermore, Johnson et al. (2014) presented evidence of reciprocal regulation of protein synthesis in the cytosol and the mitochondria of human embryonic kidney cells. The authors found that amino acid starvation led to an inhibition of mTORC1 and a decrease in cytosolic protein synthesis, whereas there was an increase in active AMPK, mitochondria density (i.e., increased citrate synthase activity), mitochondrial translation and function. Collectively, several lines of evidence support the notion that AMPK activation impairs ribosome biogenesis, and some evidence suggests that mTORC1 signaling may negatively affect certain aspects of mitochondrial biogenesis. However, the latter data are not as conclusive.

Aside from the aforementioned AMPK and mTORC1 data, sequencing data from human blood cells show ribosomal DNA (rDNA) and mitochondrial copy number (or “dose”), both of which can vary between individuals, are inversely correlated between one another (Gibbons et al., 2014). In explaining these findings, the authors suggested a tight regulatory relationship exists between rDNA abundance, the mRNA expression of ribosomal proteins, and mitochondrial DNA (mtDNA) abundance. While these data are provocative in making the case for ribosome and mitochondria competition, determining whether this relationship exists in skeletal muscle remains unknown.

Finally, ribosome biogenesis and mitochondrial biogenesis require cellular energy that is greater than metabolic homeostasis. In general, transcription and translation are ATP-consuming processes (Lynch and Marinov, 2015). The 80S ribosome contains 79 proteins and four rRNAs, and there are ~1,500 mitochondrial proteins (Boengler et al., 2011). Thus, the transcription of these components requires ATP, and the translation of mRNAs into protein requires additional ATP. It has also been suggested that ribosome assembly in eukaryotes is an energy-consuming process given that the nuclear export and assembly of the ribosome subunits involves various nucleotide-hydrolyzing enzymes (Strunk and Karbstein, 2009). Rodent studies have reported that muscle ribosomes and mitochondria exhibit rapid decay rates in response to unloading schemes (Steffen and Musacchia, 1984; Wagatsuma et al., 2011). These findings also support the notion that maintaining ribosome and mitochondrial densities are an energetic burden to muscle cells. Therefore, aside from the aforementioned mechanisms, which may contribute to the competition between ribosome and mitochondrial biogenesis, these latter points call into question as to whether or not muscle cells have the “energy bandwidth” to simultaneously promote both processes.

## What Evidence is There Suggesting Mitochondrial and Ribosome Biogenesis Do Not Compete?

To this point, we have provided evidence in favor of the biogenesis competition paradigm, in which ribosome biogenesis is prioritized with resistance training while mitochondrial biogenesis is prioritized with endurance training, or an interference effect is observed when both modes of exercise are performed concurrently. However, there is also evidence available suggesting that both processes can occur simultaneously. Tang et al. (2006), for example, reported increased muscle fiber hypertrophy and mitochondrial density (i.e., citrate synthase activity) in young males after 12weeks of resistance training, although markers of ribosomal or mitochondrial biogenesis were not assessed. Our laboratory has also reported increased citrate synthase activity after resistance training in a cohort of older participants, concomitantly with an increase in hypertrophy (Lamb et al., 2020). The same cohort of participants showed an increase in protein content of the mitochondrial electron transport chain complexes and markers of mitochondrial remodeling (Mesquita et al., 2020). However, there was no change in PGC-1a and TFAM protein content, and the activation of these signaling pathways were not interrogated. Nonetheless, our studies highlight the possibility that age and/or fitness status may play a role in the interaction between the biogenesis processes.

Notably, studies using tracer methodology to directly measure ribosome and mitochondrial biogenesis have shown that resistance training is capable of increasing both ribosome (Sieljacks et al., 2019) and mitochondrial biogenesis (Groennebaek et al., 2018). Regarding changes in the signaling pathway involved in mitochondrial adaptations, resistance training increased ACC (Ser^79^) and p38-MAPK phosphorylation, but AMPK phosphorylation remained unchanged (Groennebaek et al., 2018). Importantly, the authors reported no significant correlation between mitochondrial protein synthesis and changes in citrate synthase activity. Similarly, even though total RNA content also increased in Sieljack et al. (2019) study, the authors found no significant difference between total RNA content and RNA synthesis rate. The results of both studies suggest that resistance exercise can lead to both ribosome and mitochondrial biogenesis but reinforce the need to be careful when using measures of organelle content (i.e., citrate synthase activity and total RNA content) as an indicative of biogenesis.

In addition, concurrent training, which involves simultaneously engaging in resistance and endurance training, is a prime candidate for increasing ribosome and mitochondrial biogenesis. A landmark study by Hickson (1980) showed that concurrent training interfered with strength and hypertrophy adaptations when compared with resistance training alone. However, a comprehensive review by Fyfe et al. (2014) challenges the notion as to whether concurrent training interferes with resistance training adaptations. Moreover, a series of meta-analyses (Denadai et al., 2017; Murlasits et al., 2018; Sabag et al., 2018) suggest the interference effect elicited through endurance training is contextual and depends on factors such as endurance training modality (e.g., run training vs. cycle training) as well as endurance training frequency and duration. A number of other variables can be manipulated and potentially affect the outcome, including the training timing, which mode of training is done first, the time between the two bouts (hours or days), and whether nutritional support is given between bouts.

Furthermore, studies show that concurrent training increases maximal aerobic capacity as well as strength and hypertrophy (Mccarthy et al., 1995; Balabinis et al., 2003; Sillanpaa et al., 2008; Lundberg et al., 2014). These studies did not determine if phenotypic changes coincided with increased mitochondrial and ribosome biogenesis. However, Fyfe and colleagues have published two reports suggesting concurrent resistance training and high-intensity interval training may increase both processes. The first study (Fyfe et al., 2016) showed that compared with resistance exercise only, high-intensity interval training and resistance exercise enhanced ACC phosphorylation (Ser^79^; a readout of AMPK activity), *PPARGC1A* mRNA expression (suggestive of increased mitochondrial biogenesis), and mTOR phosphorylation [Ser^2448^; which may indicate enhanced mTOR activity, although this has been debated (Figueiredo et al., 2017)]. The second study by Fyfe et al. (2018) involved three groups of participants who undertook resistance training only, high-intensity interval training + resistance training, or moderate-intensity continuous training + resistance training for 8weeks. Following the training intervention, basal 45S pre-rRNA, 28S rRNA, and 5.8S rRNA expression were greater in the two groups that incorporated high-intensity interval training or moderate-intensity continuous training vs. resistance training alone. Total RNA per mg tissue also increased in the high-intensity interval training + resistance training, or moderate-intensity continuous training + resistance groups by ~20–30%, albeit these increases were not statistically significant. Lundberg et al. (2014) have also reported that 5weeks of concurrent training increases quadriceps hypertrophy (+6%), endurance performance (+22%), and muscle citrate synthase activity (+18%).

Moreover, the order of exercise (resistance exercise followed by endurance exercise or the opposite) is an important variable in concurrent studies. Wang et al. (2011) showed that performing a bout of resistance exercise after endurance exercise enhanced the signaling cascade for mitochondrial biogenesis. The authors found a concomitant activation of AMPK and mTOR and an increased expression of PGC-1a and PGC-1-related coactivator (PRC). However, markers of ribosome biogenesis were not examined making it difficult to determine if mitochondrial and ribosome biogenesis coincided. Apro et al. (2013), on the other hand, investigated the effects of performing endurance exercise after resistance exercise on mTORC1 and AMPK signaling pathways. Activation of the mTORC1 by resistance exercise was not impaired by subsequent concurrent endurance exercise. However, the authors found that phosphorylation of AMPK was decreased 3h after both resistance exercise-only and concurrent exercise, suggesting that prior activation of mTORC may suppress AMPK activation.

Beyond concurrent training, it is possible that other types of training, such as low-load blood flow restricted or low-load/high-volume resistance training to failure may simultaneously enhance mitochondrial and ribosome biogenesis. The studies of Groeenebaek et al. and Sieljacks et al. cited previously found that low-load blood flow restricted resistance training increased both mitochondrial (Groennebaek et al., 2018) and ribosome biogenesis (Sieljacks et al., 2019), with no difference when compared to a high-load resistance training. Furthermore, low-load/high-volume resistance training paradigm can assume several forms, but the most studied paradigm involves participants performing sets at 30% 1RM to failure (30FAIL; Mitchell et al., 2012; Jenkins et al., 2016, 2017; Morton et al., 2016, 2019; Haun et al., 2017). Lim et al. (2019) recently published a study which compared three groups of participants who trained for 10weeks (3days/week) with either 80FAIL, 30FAIL, or 30% 1RM loads, which were volume-matched to the 80FAIL group. While the authors did not report significant changes in mitochondrial volume markers (i.e., cytochrome C and COX IV protein levels), robust alterations in these markers occurred in the 30FAIL group. Markers of mitochondrial remodeling (i.e., PARKIN, OPA1, and FIS1 protein levels) also increased only in the 30FAIL group. Indeed, this evidence suggests mitochondrial biogenesis may have increased in the 30FAIL group, albeit markers of ribosome biogenesis were not assessed. Nonetheless, muscle hypertrophy did occur in the 30FAIL group. Thus, considering these studies, it seems plausible that 30% 1RM resistance training to failure may enhance mitochondrial and ribosome biogenesis.

## Other Considerations to the Competition Paradigm

A major limitation to the biogenesis competition paradigm is that AMPK and mTORC1 is primarily responsible for said competition. If this is indeed the case, then the paradigm would likely have to operate through an AMPK-mTORC1 signaling “switch” in response to each form of training. This switch has been proposed to occur in the skeletal muscle of rats following prolonged low-frequency stimulation vs. short bursts of high-frequency stimulation (Atherton et al., 2005). However, the acute post-exercise time course data regarding AMPK and mTORC1 activity in humans are more nuanced. For instance, Dreyer et al. (2006) reported that one bout of unaccustomed resistance exercise concomitantly increases AMPK activity and mTORC1 signaling markers 2hours post-exercise. Likewise, Mascher et al. (2011) reported that a 60-min cycling bout concomitantly increases AMPK activity and mTORC1 signaling markers 2hours post-exercise. The study by Wilkinson et al. (2008) similarly demonstrated that a bout of unilateral resistance and endurance training increased the phosphorylation of AMPK (Thr^172^) immediately following exercise. According to the competition paradigm, these findings suggest that resistance and endurance exercise should initiate mitochondrial and ribosomal biogenesis.

**Figure 1:**
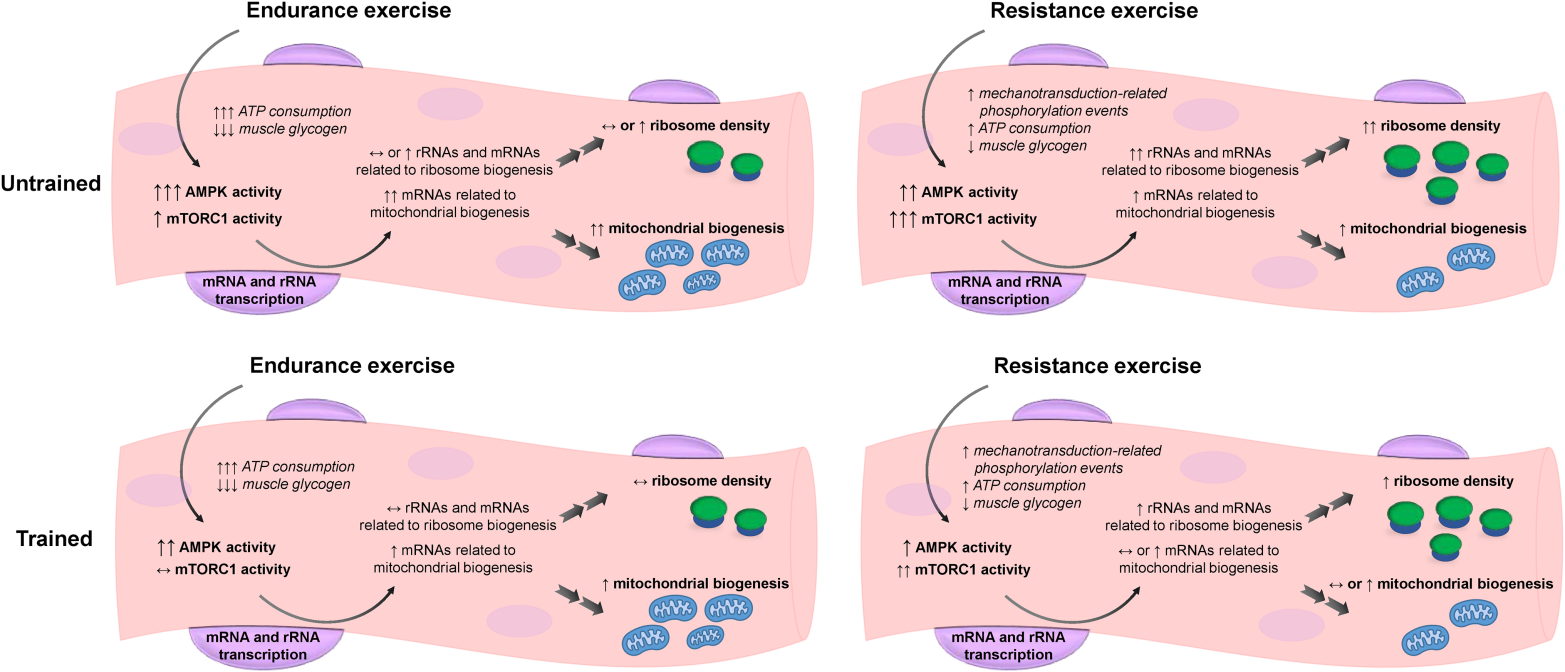
Hypothetical representation of ribosome and mitochondrial biogenesis and the respective signaling responses to endurance and resistance exercise in untrained and trained states. Untrained individuals present a more generic response to exercise, with increases in both 5' AMP-activated protein kinase (AMPK) and mechanistic target of rapamycin complex 1 (mTORC1) signaling with either endurance or resistance exercise, which may ultimately lead to a concomitant increase in ribosome and mitochondrial biogenesis. Upon training, the signaling responses to exercise seem to be attenuated and mitochondrial biogenesis is prioritized with endurance training, while ribosome biogenesis is prioritized with resistance training. Note that even in trained subjects, AMPK exhibits a more generic response to exercise compared to mTORC1.

Furthermore, Coffey et al. (2006) explored the effects of training status and accustomization to different exercise training modes (resistance vs. endurance exercise) and their data illustrate the complexity of the signaling AMPK and mTORC pathways response to exercise. The authors had a group of endurance trained and a group of resistance trained individuals perform one bout of endurance exercise and one bout of resistance exercise on different sessions. Their results suggest that untrained individuals might present a more generic response to exercise, with increases in both signaling pathways with either endurance or resistance exercise. However, as one becomes more accustomed to an exercise mode through training, the signaling responses to exercise seem to be attenuated. Moreover, AMPK signaling seems to be less specific, being activated with both endurance and resistance exercises, while mTORC is preferentially activated in response to resistance exercise (Vissing et al., 2013). In addition, considering that the response to exercise in untrained subjects seems to be fairly generic, performing concurrent training instead of resistance-only or endurance-only exercise could have an additive instead of an interference effect. This is supported by the work of Wang et al. (2011), which showed that performing resistance exercise after cycling enhanced markers of mitochondrial adaptations compared to cycling-only. However, this effect is likely dependent on a myriad of other factors, such as interval between exercise bouts and the volume of each differentiated exercise mode. More studies specifically designed to answer that question are warranted. The illustration in Figure 1 summarizes how AMPK and mTORC1 crosstalk during and following bouts of endurance and resistance exercise facilitates mitochondrial and ribosome biogenesis, respectively. It should be noted, however, that endurance exercise does not exclusively activate AMPK and inhibit mTORC1 signaling. Likewise, resistance exercise does not exclusively activate mTORC1 signaling and de-activate AMPK.

Moreover, the timing of skeletal muscle biopsies and therefore of the measurements of AMPK/mTORC1 activation is commonly referred as a limitation and a possible source of inconsistencies found between different studies (Gibala et al., 2009; Figueiredo et al., 2015; Stec et al., 2015). Besides establishing the time-course of activation, the exact timing of measurements can give important information regarding the interplay between AMPK and mTORC signaling pathways. There is evidence to suggest that even though both mTORC1 and AMPK can be activated in response to resistance exercise, mTORC1 is activated once AMPK signaling subsides (Vissing et al., 2013). Similar findings have been reported with an *ex-vivo* endurance exercise model (Jakobsgaard et al., 2021). In addition, Vissing et al. (2013) results showed that mTORC1 peak activation was at 5h post-exercise and remained upregulated until 22h pos-exercise. Therefore, if studies do not collect muscle tissue in several time-points (e.g., only perform biopsies 1h and/or 3h post-exercise), this important information about the signaling response to exercise could be missed. However, it is important to note that as previously mentioned, other studies have shown that AMPK and mTORC1 can be concomitantly activated (Wang et al., 2011; Fyfe et al., 2016).

Data from both animal and *in vitro* models also challenge the AMPK-mTORC1 switch theory. Drake et al. (2013) demonstrated a null effect of mTOR inhibition on mitochondrial biogenesis markers in mice fed a rapamycin-supplemented diet for 12weeks. There are also *in vitro* data suggesting mTOR signaling enhances mitochondrial biogenesis (Morita et al., 2013). Likewise, a review by Morita et al. (2015) provides several lines of evidence to suggest mTORC1 enhances mitochondrial function through the increased translation of transcription factors that regulate the expression of nuclear-encoded mitochondrial genes. An elegant study performed by Cunningham et al. (2007) demonstrated through a series of experiments that mTOR is necessary for proper mitochondrial oxidative function and biogenesis. The authors found that inhibition of mTOR by rapamycin decreased the expression of important mitochondrial transcription factors, gene targets of PGC-1α, and mitochondrial respiration in C_2_C_12_ myotubes. In addition, mice exposed to the same treatment also experienced similar effects. The authors proceeded with additional experiments to show that mTOR-dependent regulation of mitochondrial biogenesis and function is achieved through direct modulation of YY1-PGC-1α.

Furthermore, it is notable that mTOR complex 2 (mTORC2) is also involved with mitochondrial physiology. The differences between the mTORC1 and mTORC2 complexes are subtle; specifically, mTORC2 contains the mTOR, Rictor, LST8 and SIN1 proteins (Loewith et al., 2002). Whereas mTORC1 functions as a nutrient/amino acid sensing complex, mTORC2 receives intracellular signals from extracellular growth factor binding (Jhanwar-Uniyal et al., 2019). Interestingly, data suggest that mTORC2 stimulates mitochondrial biogenesis in liver (Betz et al., 2013) and myeloid dendritic cells (Watson et al., 2019), although equivocal data exist in macrophages from Rictor-knockout mice (Oh et al., 2017). Studies examining mTORC2 activity responses to exercise bouts or training are sparse relative to studies examining mTORC1 responses. However, evidence suggests that mTORC2 activity increases in response to an endurance bout in rodents (Kleinert et al., 2017). In contrast, skeletal muscle mTORC2 activity seems unresponsive to a bout of resistance training in humans based upon the localization of the complex not being altered following a bout of resistance training (Hodson et al., 2017). These data add to the proposed competition paradigm in that mTOR may be involved in both biogenesis processes depending upon whether mTORC1 or mTORC2 is stimulated. However, again, more studies are needed before definitive conclusions can be drawn.

Additionally, we have previously made the case that both ribosome and mitochondrial biogenesis are metabolically demanding processes and that maintaining a high density of both organelles would place an energetic demand on the cells. However, as mitochondria are the main energy-producing organelles in the cell, it could also be argued that it is counterintuitive to decrease its density when the cell is facing an increased energy demand, such as during increased ribosome biogenesis and cytosolic protein synthesis after resistance exercise. Moreover, several proteins needed for mitochondrial biogenesis are encoded in nuclear DNA and synthesized by cytosolic ribosomes before they can be imported into the mitochondria (Jornayvaz and Shulman, 2010; Perry and Hawley, 2018). Again, it would be counterintuitive to decrease ribosome density when there is an increased demand for nuclear-encoded proteins needed for mitochondrial biogenesis. Therefore, ribosome and mitochondrial biogenesis would be expected to be closely related processes.

## Conclusion

There is compelling evidence suggesting that competition between ribosomal and mitochondrial biogenesis does not exist. Several studies have shown results suggesting that both processes can occur simultaneously in response to different types of exercise (Dreyer et al., 2006; Tang et al., 2006; Mascher et al., 2011; Fyfe et al., 2016; Lamb et al., 2020). Evidence from studies using tracer methodology especially indicate that resistance training is capable of inducing both mitochondrial (Groennebaek et al., 2018) and ribosome (Sieljacks et al., 2019) biogenesis. Further, evidence for mTOR regulation of mitochondrial biogenesis (Cunningham et al., 2007) highlights that the interaction between AMPK and mTORC signaling pathways is more complex than initially thought. Therefore, it is likely that instead of a competition between the two processes, what happens is an exercise mode-specific response, where endurance exercise stimulates AMPK signaling more so than mTORC1 signaling during periods of recovery between exercise bouts, and resistance exercise stimulates the opposite phenomena. There is also the possibility that other unidentified signaling mediators increase following bouts of resistance and endurance exercise that interfere with mitochondrial and ribosome biogenesis, respectively. In this regard, −omics-based investigations may be fruitful in uncovering these potential targets if said targets exist.

Given the evidence cited in this review, it is pragmatic for individuals who seek to enhance muscle hypertrophy and aerobic capacity to engage in concurrent training. However, whether concurrent training accomplishes these adaptations through increased mitochondrial and ribosome biogenesis remains to be fully elucidated. The available data suggesting 30FAIL and blood flow restricted resistance training can enhance both processes is also compelling, and future studies examining this possibility are warranted. Importantly, more studies utilizing tracer methodology to directly assess both mitochondrial and ribosome biogenesis are needed. Research examining the interplay between the mitochondrial and ribosome biogenesis responses to exercise training will ultimately augment our understanding of skeletal muscle physiology. Critically, such research will be fruitful for individuals seeking to apply this knowledge in applied settings.

## Author Contributions

PM, CV, SP, JM, KY, AK, and MR drafted the manuscript. PM and MR generated the figures. All authors reviewed and approved the final manuscript.

## Funding

No funding was received for this article. APC costs were paid by Auburn University.

## Conflict of Interest

The authors declare that this article was written in the absence of any commercial or financial relationships that could be construed as a potential conflict of interest.

## Publisher’s Note

All claims expressed in this article are solely those of the authors and do not necessarily represent those of their affiliated organizations, or those of the publisher, the editors and the reviewers. Any product that may be evaluated in this article, or claim that may be made by its manufacturer, is not guaranteed or endorsed by the publisher.

## References

[ref1] Apro W. Wang L. Ponten M. Blomstrand E. Sahlin K. (2013). Resistance exercise induced mTORC1 signaling is not impaired by subsequent endurance exercise in human skeletal muscle. Am. J. Physiol. Endocrinol. Metab. 305, E22–E32. doi: 10.1152/ajpendo.00091.2013, PMID: 23632629

[ref2] Atherton P. J. Babraj J. Smith K. Singh J. Rennie M. J. Wackerhage H. (2005). Selective activation of AMPK-PGC-1alpha or PKB-TSC2-mTOR signaling can explain specific adaptive responses to endurance or resistance training-like electrical muscle stimulation. FASEB J. 19, 786–788. doi: 10.1096/fj.04-2179fje, PMID: 15716393

[ref3] Balabinis C. P. Psarakis C. H. Moukas M. Vassiliou M. P. Behrakis P. K. (2003). Early phase changes by concurrent endurance and strength training. J. Strength Cond. Res. 17, 393–401. doi: 10.1519/1533-4287(2003)017<0393:EPCBCE>2.0.CO;2, PMID: 12741884

[ref4] Bamman M. M. Roberts B. M. Adams G. R. (2018). Molecular regulation of exercise-induced muscle fiber hypertrophy. Cold Spring Harb. Perspect. Med. 8:e029751. doi: 10.1101/cshperspect.a029751, PMID: 28490543 PMC5983156

[ref5] Betz C. Stracka D. Prescianotto-Baschong C. Frieden M. Demaurex N. Hall M. N. (2013). Feature article: mTOR complex 2-Akt signaling at mitochondria-associated endoplasmic reticulum membranes (MAM) regulates mitochondrial physiology. Proc. Natl. Acad. Sci. 110, 12526–12534. doi: 10.1073/pnas.1302455110, PMID: 23852728 PMC3732980

[ref6] Bodine S. C. Stitt T. N. Gonzalez M. Kline W. O. Stover G. L. Bauerlein R.. (2001). Akt/mTOR pathway is a crucial regulator of skeletal muscle hypertrophy and can prevent muscle atrophy in vivo. Nat. Cell Biol. 3, 1014–1019. doi: 10.1038/ncb1101-1014, PMID: 11715023

[ref7] Boengler K. Heusch G. Schulz R. (2011). Nuclear-encoded mitochondrial proteins and their role in cardioprotection. Biochim. Biophys. Acta 1813, 1286–1294. doi: 10.1016/j.bbamcr.2011.01.009, PMID: 21255616

[ref8] Bouchez C. Devin A. (2019). Mitochondrial biogenesis and mitochondrial reactive oxygen species (ROS): a complex relationship regulated by the cAMP/PKA Signaling pathway. Cell 8:287. doi: 10.3390/cells8040287, PMID: 30934711 PMC6523352

[ref9] Brook M. S. Wilkinson D. J. Mitchell W. K. Lund J. L. Phillips B. E. Szewczyk N. J.. (2017). A novel D2O tracer method to quantify RNA turnover as a biomarker of de novo ribosomal biogenesis, in vitro, in animal models, and in human skeletal muscle. Am. J. Physiol. Endocrinol. Metab. 313, E681–E689. doi: 10.1152/ajpendo.00157.2017, PMID: 28811296 PMC5814597

[ref10] Burgomaster K. A. Howarth K. R. Phillips S. M. Rakobowchuk M. Macdonald M. J. Mcgee S. L.. (2008). Similar metabolic adaptations during exercise after low volume sprint interval and traditional endurance training in humans. J. Physiol. 586, 151–160. doi: 10.1113/jphysiol.2007.142109, PMID: 17991697 PMC2375551

[ref11] Cao Y. Bojjireddy N. Kim M. Li T. Zhai P. Nagarajan N.. (2017). Activation of gamma2-AMPK suppresses ribosome biogenesis and protects against myocardial ischemia/reperfusion injury. Circ. Res. 121, 1182–1191. doi: 10.1161/CIRCRESAHA.117.311159, PMID: 28835357 PMC5659937

[ref12] Chaillou T. Kirby T. J. Mccarthy J. J. (2014). Ribosome biogenesis: emerging evidence for a central role in the regulation of skeletal muscle mass. J. Cell. Physiol. 229, 1584–1594. doi: 10.1002/jcp.24604, PMID: 24604615 PMC4868551

[ref13] Chiao Y. A. Kolwicz S. C. Basisty N. Gagnidze A. Zhang J. Gu H.. (2016). Rapamycin transiently induces mitochondrial remodeling to reprogram energy metabolism in old hearts. Aging 8, 314–327. doi: 10.18632/aging.100881, PMID: 26872208 PMC4789585

[ref14] Choi Y. J. Park Y. J. Park J. Y. Jeong H. O. Kim D. H. Ha Y. M.. (2012). Inhibitory effect of mTOR activator MHY1485 on autophagy: suppression of lysosomal fusion. PLoS One 7:e43418. doi: 10.1371/journal.pone.0043418, PMID: 22927967 PMC3425474

[ref15] Cochran A. J. Percival M. E. Tricarico S. Little J. P. Cermak N. Gillen J. B.. (2014). Intermittent and continuous high-intensity exercise training induce similar acute but different chronic muscle adaptations. Exp. Physiol. 99, 782–791. doi: 10.1113/expphysiol.2013.077453, PMID: 24532598

[ref16] Coffey V. G. Zhong Z. Shield A. Canny B. J. Chibalin A. V. Zierath J. R.. (2006). Early signaling responses to divergent exercise stimuli in skeletal muscle from well-trained humans. FASEB J. 20, 190–192. doi: 10.1096/fj.05-4809fje, PMID: 16267123

[ref17] Correll C. C. Bartek J. Dundr M. (2019). The nucleolus: a multiphase condensate balancing ribosome synthesis and translational capacity in health, aging and ribosomopathies. Cells 8:869. doi: 10.3390/cells8080869, PMID: 31405125 PMC6721831

[ref18] Costill D. L. Fink W. J. Pollock M. L. (1976). Muscle fiber composition and enzyme activities of elite distance runners. Med. Sci. Sports 8, 96–100. PMID: . 957938

[ref19] Cunningham J. T. Rodgers J. T. Arlow D. H. Vazquez F. Mootha V. K. Puigserver P. (2007). mTOR controls mitochondrial oxidative function through a YY1-PGC-1alpha transcriptional complex. Nature 450, 736–740. doi: 10.1038/nature06322, PMID: 18046414

[ref20] Deepa S. S. Walsh M. E. Hamilton R. T. Pulliam D. Shi Y. Hill S.. (2013). Rapamycin modulates markers of mitochondrial biogenesis and fatty acid oxidation in the adipose tissue of db/db mice. J Biochem Pharmacol Res 1, 114–123. PMID: . 24010023 PMC3760510

[ref21] Denadai B. S. De Aguiar R. A. De Lima L. C. Greco C. C. Caputo F. (2017). Explosive training and heavy weight training are effective for improving running economy in endurance athletes: a systematic review and meta-analysis. Sports Med. 47, 545–554. doi: 10.1007/s40279-016-0604-z, PMID: 27497600

[ref22] Derenzini M. Montanaro L. Trere D. (2017). Ribosome biogenesis and cancer. Acta Histochem. 119, 190–197. doi: 10.1016/j.acthis.2017.01.009, PMID: 28168996

[ref23] Drake J. C. Peelor F. F. Biela L. M. Watkins M. K. Miller R. A. Hamilton K. L.. (2013). Assessment of mitochondrial biogenesis and mTORC1 signaling during chronic rapamycin feeding in male and female mice. J Gerontol A Biol Sci Med Sci. 68, 1493–1501. 23657975 10.1093/gerona/glt047 PMC3814233

[ref24] Dreyer H. C. Fujita S. Cadenas J. G. Chinkes D. L. Volpi E. Rasmussen B. B. (2006). Resistance exercise increases AMPK activity and reduces 4E-BP1 phosphorylation and protein synthesis in human skeletal muscle. J. Physiol. 576, 613–624. doi: 10.1113/jphysiol.2006.113175, PMID: 16873412 PMC1890364

[ref25] Drummond M. J. Fry C. S. Glynn E. L. Dreyer H. C. Dhanani S. Timmerman K. L.. (2009). Rapamycin administration in humans blocks the contraction-induced increase in skeletal muscle protein synthesis. J. Physiol. 587, 1535–1546. doi: 10.1113/jphysiol.2008.163816, PMID: 19188252 PMC2678224

[ref26] Figueiredo V. C. Caldow M. K. Massie V. Markworth J. F. Cameron-Smith D. Blazevich A. J. (2015). Ribosome biogenesis adaptation in resistance training-induced human skeletal muscle hypertrophy. Am. J. Physiol. Endocrinol. Metab. 309, E72–E83. doi: 10.1152/ajpendo.00050.2015, PMID: 25968575

[ref27] Figueiredo V. C. Markworth J. F. Cameron-Smith D. (2017). Considerations on mTOR regulation at serine 2448: implications for muscle metabolism studies. Cell. Mol. Life Sci. 74, 2537–2545. doi: 10.1007/s00018-017-2481-5, PMID: 28220207 PMC11107628

[ref28] Figueiredo V. C. Mccarthy J. J. (2019). Regulation of ribosome biogenesis in skeletal muscle hypertrophy. Physiol. (Bethesda) 34, 30–42. doi: 10.1249/JES.0000000000000179, PMID: 30540235 PMC6383632

[ref29] Figueiredo V. C. Wen Y. Alkner B. Fernandez-Gonzalo R. Norrbom J. Vechetti I. J. Jr.. (2021). Genetic and epigenetic regulation of skeletal muscle ribosome biogenesis with exercise. J. Physiol. 599, 3363–3384. doi: 10.1113/JP281244, PMID: 33913170

[ref30] Fujii N. Hayashi T. Hirshman M. F. Smith J. T. Habinowski S. A. Kaijser L.. (2000). Exercise induces isoform-specific increase in 5'AMP-activated protein kinase activity in human skeletal muscle. Biochem. Biophys. Res. Commun. 273, 1150–1155. doi: 10.1006/bbrc.2000.3073, PMID: 10891387

[ref31] Fyfe J. J. Bishop D. J. Bartlett J. D. Hanson E. D. Anderson M. J. Garnham A. P.. (2018). Enhanced skeletal muscle ribosome biogenesis, yet attenuated mTORC1 and ribosome biogenesis-related signalling, following short-term concurrent versus single-mode resistance training. Sci. Rep. 8, 1–21. doi: 10.1038/s41598-017-18887-6, PMID: 29330460 PMC5766515

[ref32] Fyfe J. J. Bishop D. J. Stepto N. K. (2014). Interference between concurrent resistance and endurance exercise: molecular bases and the role of individual training variables. Sports Med. 44, 743–762. doi: 10.1007/s40279-014-0162-1, PMID: 24728927

[ref33] Fyfe J. J. Bishop D. J. Zacharewicz E. Russell A. P. Stepto N. K. (2016). Concurrent exercise incorporating high-intensity interval or continuous training modulates mTORC1 signaling and microRNA expression in human skeletal muscle. Am. J. Physiol. Regul. Integr. Comp. Physiol. 310, R1297–R1311. doi: 10.1152/ajpregu.00479.2015, PMID: 27101297

[ref34] Gibala M. J. Mcgee S. L. Garnham A. P. Howlett K. F. Snow R. J. Hargreaves M. (2009). Brief intense interval exercise activates AMPK and p38 MAPK signaling and increases the expression of PGC-1alpha in human skeletal muscle. J. Appl. Physiol. 106, 929–934. doi: 10.1152/japplphysiol.90880.2008, PMID: 19112161

[ref35] Gibbons J. G. Branco A. T. Yu S. Lemos B. (2014). Ribosomal DNA copy number is coupled with gene expression variation and mitochondrial abundance in humans. Nat. Commun. 5:5850. doi: 10.1038/ncomms5850, PMID: 25209200

[ref36] Goodman C. A. (2019). Role of mTORC1 in mechanically induced increases in translation and skeletal muscle mass. J Appl Physioln. 127, 581–590. doi: 10.1152/japplphysiol.01011.2018, PMID: 30676865

[ref37] Groennebaek T. Jespersen N. R. Jakobsgaard J. E. Sieljacks P. Wang J. Rindom E.. (2018). Skeletal muscle mitochondrial protein synthesis and respiration increase with low-load blood flow restricted as well as high-load resistance training. Front. Physiol. 9:1796. doi: 10.3389/fphys.2018.01796, PMID: 30618808 PMC6304675

[ref38] Groennebaek T. Vissing K. (2017). Impact of resistance training on skeletal muscle mitochondrial biogenesis, content, and function. Front. Physiol. 8:713. doi: 10.3389/fphys.2017.00713, PMID: 28966596 PMC5605648

[ref39] Gundermann D. M. Walker D. K. Reidy P. T. Borack M. S. Dickinson J. M. Volpi E.. (2014). Activation of mTORC1 signaling and protein synthesis in human muscle following blood flow restriction exercise is inhibited by rapamycin. Am. J. Physiol. Endocrinol. Metab. 306, E1198–E1204. doi: 10.1152/ajpendo.00600.2013, PMID: 24691032 PMC4116405

[ref40] Hammarstrom D. Ofsteng S. Koll L. Hanestadhaugen M. Hollan I. Apro W.. (2020). Benefits of higher resistance-training volume are related to ribosome biogenesis. J. Physiol. 598, 543–565. doi: 10.1113/JP278455, PMID: 31813190

[ref41] Hansson B. Olsen L. A. Nicoll J. X. Von Walden F. Melin M. Stromberg A.. (2019). Skeletal muscle signaling responses to resistance exercise of the elbow extensors are not compromised by a preceding bout of aerobic exercise. Am. J. Physiol. Regul. Integr. Comp. Physiol. 317, R83–R92. doi: 10.1152/ajpregu.00022.2019, PMID: 30969843

[ref42] Haun C. T. Mumford P. W. Roberson P. A. Romero M. A. Mobley C. B. Kephart W. C.. (2017). Molecular, neuromuscular, and recovery responses to light versus heavy resistance exercise in young men. Physiol. Rep. 5:e13457. doi: 10.14814/phy2.13457, PMID: 28963127 PMC5617935

[ref43] Haun C. T. Vann C. G. Mobley C. B. Osburn S. C. Mumford P. W. Roberson P. A.. (2019). Pre-training skeletal muscle fiber size and predominant Fiber type best predict hypertrophic responses to 6 weeks of resistance training in previously trained young men. Front. Physiol. 10:297. doi: 10.3389/fphys.2019.00297, PMID: 30971942 PMC6445136

[ref44] Hayase K. Yokogoshi H. (1992). Effect of exercise on tissue protein synthesis in rats. Biosci. Biotechnol. Biochem. 56, 1637–1639. doi: 10.1271/bbb.56.1637, PMID: 1369062

[ref45] Henras A. K. Plisson-Chastang C. O’donohue M. F. Chakraborty A. Gleizes P. E. (2015). An overview of pre-ribosomal RNA processing in eukaryotes. Wiley Interdiscip Rev RNA 6, 225–242. doi: 10.1002/wrna.1269, PMID: 25346433 PMC4361047

[ref46] Hickson R. C. (1980). Interference of strength development by simultaneously training for strength and endurance. Eur. J. Appl. Physiol. Occup. Physiol. 45, 255–263. doi: 10.1007/BF00421333, PMID: 7193134

[ref47] Hodson N. Mcglory C. Oikawa S. Y. Jeromson S. Song Z. Ruegg M. A.. (2017). Differential localization and anabolic responsiveness of mTOR complexes in human skeletal muscle in response to feeding and exercise. Am. J. Physiol. Cell Physiol. 313, C604–C611. doi: 10.1152/ajpcell.00176.2017, PMID: 28971834 PMC5814591

[ref48] Hoppe S. Bierhoff H. Cado I. Weber A. Tiebe M. Grummt I.. (2009). AMP-activated protein kinase adapts rRNA synthesis to cellular energy supply. Proc. Natl. Acad. Sci. 106, 17781–17786. doi: 10.1073/pnas.0909873106, PMID: 19815529 PMC2764937

[ref49] Hulmi J. J. Tannerstedt J. Selanne H. Kainulainen H. Kovanen V. Mero A. A. (2009). Resistance exercise with whey protein ingestion affects mTOR signaling pathway and myostatin in men. J. Appl. Physiol. 106, 1720–1729. doi: 10.1152/japplphysiol.00087.2009, PMID: 19299575

[ref50] Jakobsgaard J. E. Andresen J. De Paoli F. V. Vissing K. (2021). Skeletal muscle phenotype signaling with ex vivo endurance-type dynamic contractions in rat muscle. J. Appl. Physiol. 131, 45–55. doi: 10.1152/japplphysiol.00107.2021, PMID: 34043469

[ref51] Jenkins N. D. Housh T. J. Buckner S. L. Bergstrom H. C. Smith C. M. Cochrane K. C.. (2016). Four weeks of high-versus low-load resistance training to failure on the rate of torque development, electromechanical delay, and contractile twitch properties. J. Musculoskelet. Neuronal Interact. 16, 135–144. PMID: . 27282457 PMC5114356

[ref52] Jenkins N. D. M. Miramonti A. A. Hill E. C. Smith C. M. Cochrane-Snyman K. C. Housh T. J.. (2017). Greater neural adaptations following high-vs. Low-Load Resistance Training. Front Physiol 8:331. doi: 10.3389/fphys.2017.00331, PMID: 28611677 PMC5447067

[ref53] Jhanwar-Uniyal M. Wainwright J. V. Mohan A. L. Tobias M. E. Murali R. Gandhi C. D.. (2019). Diverse signaling mechanisms of mTOR complexes: mTORC1 and mTORC2 in forming a formidable relationship. Adv Biol Regul 72, 51–62. doi: 10.1016/j.jbior.2019.03.003, PMID: 31010692

[ref54] Johnson M. A. Vidoni S. Durigon R. Pearce S. F. Rorbach J. He J.. (2014). Amino acid starvation has opposite effects on mitochondrial and cytosolic protein synthesis. PLoS One 9:e93597. doi: 10.1371/journal.pone.0093597, PMID: 24718614 PMC3981720

[ref55] Jones T. W. Howatson G. Russell M. French D. N. (2013). Performance and neuromuscular adaptations following differing ratios of concurrent strength and endurance training. J. Strength Cond. Res. 27, 3342–3351. doi: 10.1519/JSC.0b013e3181b2cf39, PMID: 24270456

[ref56] Jorgensen S. B. Wojtaszewski J. F. Viollet B. Andreelli F. Birk J. B. Hellsten Y.. (2005). Effects of alpha-AMPK knockout on exercise-induced gene activation in mouse skeletal muscle. FASEB J. 19, 1146–1148. doi: 10.1096/fj.04-3144fje, PMID: 15878932

[ref57] Jornayvaz F. R. Shulman G. I. (2010). Regulation of mitochondrial biogenesis. Essays Biochem. 47, 69–84. doi: 10.1042/bse0470069, PMID: 20533901 PMC3883043

[ref58] Kadi F. Schjerling P. Andersen L. L. Charifi N. Madsen J. L. Christensen L. R.. (2004). The effects of heavy resistance training and detraining on satellite cells in human skeletal muscles. J. Physiol. 558, 1005–1012. doi: 10.1113/jphysiol.2004.065904, PMID: 15218062 PMC1665027

[ref59] Kim H. G. Guo B. Nader G. A. (2019). Regulation of ribosome biogenesis during skeletal muscle hypertrophy. Exerc. Sport Sci. Rev. 47, 91–97. doi: 10.1249/JES.0000000000000179, PMID: 30632998

[ref60] Kleinert M. Parker B. L. Fritzen A. M. Knudsen J. R. Jensen T. E. Kjobsted R.. (2017). Mammalian target of rapamycin complex 2 regulates muscle glucose uptake during exercise in mice. J. Physiol. 595, 4845–4855. doi: 10.1113/JP274203, PMID: 28464351 PMC5509878

[ref61] Kodiha M. Salimi A. Wang Y. M. Stochaj U. (2014). Pharmacological AMP kinase activators target the nucleolar organization and control cell proliferation. PLoS One 9:e88087. doi: 10.1371/journal.pone.0088087, PMID: 24498249 PMC3907577

[ref62] Kopp K. Gasiorowski J. Z. Chen D. Gilmore R. Norton J. T. Wang C.. (2007). Pol I transcription and pre-rRNA processing are coordinated in a transcription-dependent manner in mammalian cells. Mol. Biol. Cell 18, 394–403. doi: 10.1091/mbc.e06-03-0249, PMID: 17108330 PMC1783775

[ref63] Kraemer W. J. Patton J. F. Gordon S. E. Harman E. A. Deschenes M. R. Reynolds K.. (1995). Compatibility of high-intensity strength and endurance training on hormonal and skeletal muscle adaptations. J. Appl. Physiol. 78, 976–989. doi: 10.1152/jappl.1995.78.3.976, PMID: 7775344

[ref64] Kressler D. Hurt E. Bassler J. (2017). A puzzle of life: crafting ribosomal subunits. Trends Biochem. Sci. 42, 640–654. doi: 10.1016/j.tibs.2017.05.005, PMID: 28579196

[ref65] Lamb D. A. Moore J. H. Mesquita P. H. C. Smith M. A. Vann C. G. Osburn S. C.. (2020). Resistance training increases muscle NAD(+) and NADH concentrations as well as NAMPT protein levels and global sirtuin activity in middle-aged, overweight, untrained individuals. Aging 12, 9447–9460. doi: 10.18632/aging.103218, PMID: 32369778 PMC7288928

[ref66] Lim C. Kim H. J. Morton R. W. Harris R. Phillips S. M. Jeong T. S.. (2019). Resistance exercise-induced changes in muscle phenotype are load dependent. Med. Sci. Sports Exerc. 51, 2578–2585. doi: 10.1249/MSS.0000000000002088, PMID: 31306302

[ref67] Loewith R. Jacinto E. Wullschleger S. Lorberg A. Crespo J. L. Bonenfant D.. (2002). Two TOR complexes, only one of which is rapamycin sensitive, have distinct roles in cell growth control. Mol. Cell 10, 457–468. doi: 10.1016/S1097-2765(02)00636-6, PMID: 12408816

[ref68] Lundberg T. R. Fernandez-Gonzalo R. Tesch P. A. (2014). Exercise-induced AMPK activation does not interfere with muscle hypertrophy in response to resistance training in men. J. Appl. Physiol. 116, 611–620. doi: 10.1152/japplphysiol.01082.2013, PMID: 24408998

[ref69] Luthi J. M. Howald H. Claassen H. Rosler K. Vock P. Hoppeler H. (1986). Structural changes in skeletal muscle tissue with heavy-resistance exercise. Int. J. Sports Med. 7, 123–127. doi: 10.1055/s-2008-1025748, PMID: 2942497

[ref70] Lynch M. Marinov G. K. (2015). The bioenergetic costs of a gene. Proc. Natl. Acad. Sci. 112, 15690–15695. doi: 10.1073/pnas.1514974112, PMID: 26575626 PMC4697398

[ref71] Macdougall J. D. Sale D. G. Elder G. C. Sutton J. R. (1982). Muscle ultrastructural characteristics of elite powerlifters and bodybuilders. Eur. J. Appl. Physiol. Occup. Physiol. 48, 117–126. doi: 10.1007/BF00421171, PMID: 7199447

[ref72] Marin T. L. Gongol B. Zhang F. Martin M. Johnson D. A. Xiao H.. (2017). AMPK promotes mitochondrial biogenesis and function by phosphorylating the epigenetic factors DNMT1, RBBP7, and HAT1. Sci. Signal. 10:aaf7478. doi: 10.1126/scisignal.aaf7478, PMID: 28143904 PMC5830108

[ref73] Martin J. S. Padilla J. Jenkins N. T. Crissey J. M. Bender S. B. Rector R. S.. (2012). Functional adaptations in the skeletal muscle microvasculature to endurance and interval sprint training in the type 2 diabetic OLETF rat. J. Appl. Physiol. 113, 1223–1232. doi: 10.1152/japplphysiol.00823.2012, PMID: 22923508 PMC3472489

[ref74] Mascher H. Ekblom B. Rooyackers O. Blomstrand E. (2011). Enhanced rates of muscle protein synthesis and elevated mTOR signalling following endurance exercise in human subjects. Acta Physiol (Oxf.) 202, 175–184. doi: 10.1111/j.1748-1716.2011.02274.x 21385328

[ref75] Mayer C. Grummt I. (2006). Ribosome biogenesis and cell growth: mTOR coordinates transcription by all three classes of nuclear RNA polymerases. Oncogene 25, 6384–6391. doi: 10.1038/sj.onc.1209883, PMID: 17041624

[ref76] Mayhew D. L. Kim J. S. Cross J. M. Ferrando A. A. Bamman M. M. (2009). Translational signaling responses preceding resistance training-mediated myofiber hypertrophy in young and old humans. J. Appl. Physiol. 107, 1655–1662. doi: 10.1152/japplphysiol.91234.2008, PMID: 19589955 PMC2777794

[ref77] Mccarthy J. P. Agre J. C. Graf B. K. Pozniak M. A. Vailas A. C. (1995). Compatibility of adaptive responses with combining strength and endurance training. Med. Sci. Sports Exerc. 27, 429–436. PMID: . 7752872

[ref78] Mcglory C. Devries M. C. Phillips S. M. (2017). Skeletal muscle and resistance exercise training; the role of protein synthesis in recovery and remodeling. J. Appl. Physiol. 122, 541–548. doi: 10.1152/japplphysiol.00613.2016, PMID: 27742803 PMC5401959

[ref80] Mesquita P. H. C. Lamb D. A. Parry H. A. Moore J. H. Smith M. A. Vann C. G.. (2020). Acute and chronic effects of resistance training on skeletal muscle markers of mitochondrial remodeling in older adults. Physiol. Rep. 8:e14526. doi: 10.14814/phy2.14526, PMID: 32748504 PMC7399374

[ref81] Miller B. F. Hamilton K. L. (2012). A perspective on the determination of mitochondrial biogenesis. Am. J. Physiol. Endocrinol. Metab. 302, E496–E499. doi: 10.1152/ajpendo.00578.2011, PMID: 22205627 PMC3311289

[ref82] Mitchell C. J. Churchward-Venne T. A. Bellamy L. Parise G. Baker S. K. Phillips S. M. (2013). Muscular and systemic correlates of resistance training-induced muscle hypertrophy. PLoS One 8:e78636. doi: 10.1371/journal.pone.0078636, PMID: 24130904 PMC3793973

[ref83] Mitchell C. J. Churchward-Venne T. A. West D. W. Burd N. A. Breen L. Baker S. K.. (2012). Resistance exercise load does not determine training-mediated hypertrophic gains in young men. J. Appl. Physiol. 113, 71–77. doi: 10.1152/japplphysiol.00307.2012, PMID: 22518835 PMC3404827

[ref84] Mobley C. B. Fox C. D. Thompson R. M. Healy J. C. Santucci V. Kephart W. C.. (2016). Comparative effects of whey protein versus L-leucine on skeletal muscle protein synthesis and markers of ribosome biogenesis following resistance exercise. Amino Acids 48, 733–750. doi: 10.1007/s00726-015-2121-z, PMID: 26507545

[ref85] Mobley C. B. Haun C. T. Roberson P. A. Mumford P. W. Kephart W. C. Romero M. A.. (2018). Biomarkers associated with low, moderate, and high vastus lateralis muscle hypertrophy following 12 weeks of resistance training. PLoS One 13:e0195203. doi: 10.1371/journal.pone.0195203, PMID: 29621305 PMC5886420

[ref86] Morita M. Gravel S. P. Chenard V. Sikstrom K. Zheng L. Alain T.. (2013). mTORC1 controls mitochondrial activity and biogenesis through 4E-BP-dependent translational regulation. Cell Metab. 18, 698–711. doi: 10.1016/j.cmet.2013.10.001, PMID: 24206664

[ref87] Morita M. Gravel S. P. Hulea L. Larsson O. Pollak M. St-Pierre J.. (2015). mTOR coordinates protein synthesis, mitochondrial activity and proliferation. Cell Cycle 14, 473–480. doi: 10.4161/15384101.2014.991572, PMID: 25590164 PMC4615141

[ref88] Morrison P. R. Biggs R. B. Booth F. W. (1989). Daily running for 2 wk and mRNAs for cytochrome c and alpha-actin in rat skeletal muscle. Am. J. Phys. 257, C936–C939. doi: 10.1152/ajpcell.1989.257.5.C936, PMID: 2480716

[ref89] Morton R. W. Oikawa S. Y. Wavell C. G. Mazara N. Mcglory C. Quadrilatero J.. (2016). Neither load nor systemic hormones determine resistance training-mediated hypertrophy or strength gains in resistance-trained young men. J. Appl. Physiol. 121, 129–138. doi: 10.1152/japplphysiol.00154.2016, PMID: 27174923 PMC4967245

[ref90] Morton R. W. Sonne M. W. Farias Zuniga A. Mohammad I. Y. Z. Jones A. Mcglory C.. (2019). Muscle fibre activation is unaffected by load and repetition duration when resistance exercise is performed to task failure. J. Physiol. 597, 4601–4613. doi: 10.1113/JP278056, PMID: 31294822

[ref91] Murias J. M. Kowalchuk J. M. Ritchie D. Hepple R. T. Doherty T. J. Paterson D. H. (2011). Adaptations in capillarization and citrate synthase activity in response to endurance training in older and young men. J. Gerontol. A Biol. Sci. Med. Sci. 66, 957–964. doi: 10.1093/gerona/glr096, PMID: 21715648

[ref92] Murlasits Z. Kneffel Z. Thalib L. (2018). The physiological effects of concurrent strength and endurance training sequence: A systematic review and meta-analysis. J. Sports Sci. 36, 1212–1219. doi: 10.1080/02640414.2017.1364405, PMID: 28783467

[ref93] Nader G. A. Mcloughlin T. J. Esser K. A. (2005). mTOR function in skeletal muscle hypertrophy: increased ribosomal RNA via cell cycle regulators. Am. J. Physiol. Cell Physiol. 289, C1457–C1465. doi: 10.1152/ajpcell.00165.2005, PMID: 16079186

[ref94] Oakhill J. S. Steel R. Chen Z. P. Scott J. W. Ling N. Tam S.. (2011). AMPK is a direct adenylate charge-regulated protein kinase. Science 332, 1433–1435. doi: 10.1126/science.1200094, PMID: 21680840

[ref95] Oh M. H. Collins S. L. Sun I. H. Tam A. J. Patel C. H. Arwood M. L.. (2017). mTORC2 Signaling selectively regulates the generation and function of tissue-resident peritoneal macrophages. Cell Rep. 20, 2439–2454. doi: 10.1016/j.celrep.2017.08.046, PMID: 28877476 PMC5659290

[ref96] O’neill H. M. Maarbjerg S. J. Crane J. D. Jeppesen J. Jorgensen S. B. Schertzer J. D.. (2011). AMP-activated protein kinase (AMPK) beta1beta2 muscle null mice reveal an essential role for AMPK in maintaining mitochondrial content and glucose uptake during exercise. Proc. Natl. Acad. Sci. 108, 16092–16097. doi: 10.1073/pnas.1105062108, PMID: 21896769 PMC3179037

[ref97] Parise G. Brose A. N. Tarnopolsky M. A. (2005). Resistance exercise training decreases oxidative damage to DNA and increases cytochrome oxidase activity in older adults. Exp. Gerontol. 40, 173–180. doi: 10.1016/j.exger.2004.09.002, PMID: 15763394

[ref98] Pelletier J. Thomas G. Volarevic S. (2018). Ribosome biogenesis in cancer: new players and therapeutic avenues. Nat. Rev. Cancer 18, 51–63. doi: 10.1038/nrc.2017.104, PMID: 29192214

[ref99] Perry C. G. R. Hawley J. A. (2018). Molecular basis of exercise-induced skeletal muscle mitochondrial biogenesis: historical advances, current knowledge, and future challenges. Cold Spring Harb. Perspect. Med. 8:a029686. doi: 10.1101/cshperspect.a029686, PMID: 28507194 PMC6120690

[ref100] Porter C. Reidy P. T. Bhattarai N. Sidossis L. S. Rasmussen B. B. (2015). Resistance exercise training alters mitochondrial function in human skeletal muscle. Med. Sci. Sports Exerc. 47, 1922–1931. doi: 10.1249/MSS.0000000000000605, PMID: 25539479 PMC4478283

[ref101] Reidy P. T. Borack M. S. Markofski M. M. Dickinson J. M. Fry C. S. Deer R. R.. (2017). Post-absorptive muscle protein turnover affects resistance training hypertrophy. Eur. J. Appl. Physiol. 117, 853–866. doi: 10.1007/s00421-017-3566-4, PMID: 28280974 PMC5389914

[ref102] Reznick R. M. Zong H. Li J. Morino K. Moore I. K. Yu H. J.. (2007). Aging-associated reductions in AMP-activated protein kinase activity and mitochondrial biogenesis. Cell Metab. 5, 151–156. doi: 10.1016/j.cmet.2007.01.008, PMID: 17276357 PMC1885964

[ref103] Richter E. A. Ruderman N. B. (2009). AMPK and the biochemistry of exercise: implications for human health and disease. Biochem. J. 418, 261–275. doi: 10.1042/BJ20082055, PMID: 19196246 PMC2779044

[ref104] Roberts M. D. Haun C. T. Mobley C. B. Mumford P. W. Romero M. A. Roberson P. A.. (2018a). Physiological differences Between low versus high skeletal muscle hypertrophic responders to resistance exercise training: current perspectives and future research directions. Front. Physiol. 9:834. doi: 10.3389/fphys.2018.00834, PMID: 30022953 PMC6039846

[ref105] Roberts M. D. Romero M. A. Mobley C. B. Mumford P. W. Roberson P. A. Haun C. T.. (2018b). Skeletal muscle mitochondrial volume and myozenin-1 protein differences exist between high versus low anabolic responders to resistance training. PeerJ 6:e5338. doi: 10.7717/peerj.5338, PMID: 30065891 PMC6065464

[ref106] Romero M. A. Mobley C. B. Linden M. A. Meers G. M. Martin J. S. Young K. C.. (2017). Endurance training lowers ribosome density despite increasing ribosome biogenesis markers in rodent skeletal muscle. BMC. Res. Notes 10, 1–8. doi: 10.1186/s13104-017-2736-0, PMID: 28800772 PMC5553677

[ref107] Roque W. Cuevas-Mora K. Romero F. (2020). Mitochondrial quality control in age-related pulmonary fibrosis. Int. J. Mol. Sci. 21:643. doi: 10.3390/ijms21020643, PMID: 31963720 PMC7013724

[ref108] Ruderman N. B. Park H. Kaushik V. K. Dean D. Constant S. Prentki M.. (2003). AMPK as a metabolic switch in rat muscle, liver and adipose tissue after exercise. Acta Physiol. Scand. 178, 435–442. doi: 10.1046/j.1365-201X.2003.01164.x, PMID: 12864749

[ref109] Ryan M. T. Hoogenraad N. J. (2007). Mitochondrial-nuclear communications. Annu. Rev. Biochem. 76, 701–722. doi: 10.1146/annurev.biochem.76.052305.091720, PMID: 17227225

[ref110] Sabag A. Najafi A. Michael S. Esgin T. Halaki M. Hackett D. (2018). The compatibility of concurrent high intensity interval training and resistance training for muscular strength and hypertrophy: a systematic review and meta-analysis. J. Sports Sci. 36, 2472–2483. doi: 10.1080/02640414.2018.1464636, PMID: 29658408

[ref111] Saxton R. A. Sabatini D. M. (2017). mTOR Signaling in growth, metabolism, and disease. Cell 169, 361–371. doi: 10.1016/j.cell.2017.03.035, PMID: 28388417

[ref112] Shaw R. J. (2009). LKB1 and AMP-activated protein kinase control of mTOR signalling and growth. Acta Physiol (Oxf.) 196, 65–80. doi: 10.1111/j.1748-1716.2009.01972.x, PMID: 19245654 PMC2760308

[ref113] Sieljacks P. Wang J. Groennebaek T. Rindom E. Jakobsgaard J. E. Herskind J.. (2019). Six weeks of low-load blood flow restricted and high-load resistance exercise training produce similar increases in cumulative myofibrillar protein synthesis and ribosomal biogenesis in healthy males. Front. Physiol. 10:649. doi: 10.3389/fphys.2019.00649, PMID: 31191347 PMC6548815

[ref114] Sillanpaa E. Hakkinen A. Nyman K. Mattila M. Cheng S. Karavirta L.. (2008). Body composition and fitness during strength and/or endurance training in older men. Med. Sci. Sports Exerc. 40, 950–958. doi: 10.1249/MSS.0b013e318165c854, PMID: 18408601

[ref115] Sin J. Andres A. M. Taylor D. J. Weston T. Hiraumi Y. Stotland A.. (2016). Mitophagy is required for mitochondrial biogenesis and myogenic differentiation of C2C12 myoblasts. Autophagy 12, 369–380. doi: 10.1080/15548627.2015.1115172, PMID: 26566717 PMC4836019

[ref116] Stec M. J. Kelly N. A. Many G. M. Windham S. T. Tuggle S. C. Bamman M. M. (2016). Ribosome biogenesis may augment resistance training-induced myofiber hypertrophy and is required for myotube growth in vitro. Am. J. Physiol. Endocrinol. Metab. 310, E652–E661. doi: 10.1152/ajpendo.00486.2015, PMID: 26860985 PMC4835943

[ref117] Stec M. J. Mayhew D. L. Bamman M. M. (2015). The effects of age and resistance loading on skeletal muscle ribosome biogenesis. J. Appl. Physiol. 119, 851–857. doi: 10.1152/japplphysiol.00489.2015, PMID: 26294750 PMC4747892

[ref118] Steffen J. M. Musacchia X. J. (1984). Effect of hypokinesia and hypodynamia on protein, RNA, and DNA in rat hindlimb muscles. Am. J. Phys. 247, R728–R732. doi: 10.1152/ajpregu.1984.247.4.R728, PMID: 6208790

[ref119] Steinberg G. R. Watt M. J. Mcgee S. L. Chan S. Hargreaves M. Febbraio M. A.. (2006). Reduced glycogen availability is associated with increased AMPKalpha2 activity, nuclear AMPKalpha2 protein abundance, and GLUT4 mRNA expression in contracting human skeletal muscle. Appl. Physiol. Nutr. Metab. 31, 302–312. doi: 10.1139/h06-003, PMID: 16770359

[ref120] Strunk B. S. Karbstein K. (2009). Powering through ribosome assembly. RNA 15, 2083–2104. doi: 10.1261/rna.1792109, PMID: 19850913 PMC2779681

[ref121] Tang J. E. Hartman J. W. Phillips S. M. (2006). Increased muscle oxidative potential following resistance training induced fibre hypertrophy in young men. Appl. Physiol. Nutr. Metab. 31, 495–501. doi: 10.1139/h06-026, PMID: 17111003

[ref122] Teran-Garcia M. Rankinen T. Koza R. A. Rao D. C. Bouchard C. (2005). Endurance training-induced changes in insulin sensitivity and gene expression. Am. J. Physiol. Endocrinol. Metab. 288, E1168–E1178. doi: 10.1152/ajpendo.00467.2004, PMID: 15687108

[ref123] Terzis G. Georgiadis G. Stratakos G. Vogiatzis I. Kavouras S. Manta P.. (2008). Resistance exercise-induced increase in muscle mass correlates with p70S6 kinase phosphorylation in human subjects. Eur. J. Appl. Physiol. 102, 145–152. doi: 10.1007/s00421-007-0564-y, PMID: 17874120

[ref124] Tesch P. A. Komi P. V. Hakkinen K. (1987). Enzymatic adaptations consequent to long-term strength training. Int. J. Sports Med. 8(Suppl. 1), 66–69. doi: 10.1055/s-2008-1025706, PMID: 2953691

[ref125] Tiku V. Antebi A. (2018). Nucleolar function in lifespan regulation. Trends Cell Biol. 28, 662–672. doi: 10.1016/j.tcb.2018.03.007, PMID: 29779866

[ref126] Vanderveen B. N. Fix D. K. Carson J. A. (2017). Disrupted skeletal muscle mitochondrial dynamics, Mitophagy, and biogenesis during cancer cachexia: a role for inflammation. Oxidative Med. Cell. Longev. 2017:3292087. doi: 10.1155/2017/3292087, PMID: 28785374 PMC5530417

[ref127] Vigelso A. Andersen N. B. Dela F. (2014). The relationship between skeletal muscle mitochondrial citrate synthase activity and whole body oxygen uptake adaptations in response to exercise training. Int. J. Physiol. Pathophysiol. Pharmacol. 6, 84–101. PMID: . 25057335 PMC4106645

[ref128] Vissing K. Mcgee S. Farup J. Kjolhede T. Vendelbo M. Jessen N. (2013). Differentiated mTOR but not AMPK signaling after strength vs endurance exercise in training-accustomed individuals. Scand. J. Med. Sci. Sports 23, 355–366. doi: 10.1111/j.1600-0838.2011.01395.x, PMID: 23802289

[ref129] Von Walden F. (2019). Ribosome biogenesis in skeletal muscle: coordination of transcription and translation. J. Appl. Physiol. 127, 591–598. doi: 10.1152/japplphysiol.00963.2018 31219775

[ref130] Von Walden F. Liu C. Aurigemma N. Nader G. A. (2016). mTOR signaling regulates myotube hypertrophy by modulating protein synthesis, rDNA transcription, and chromatin remodeling. Am. J. Physiol. Cell Physiol. 311, C663–C672. doi: 10.1152/ajpcell.00144.2016 27581648

[ref131] Wagatsuma A. Kotake N. Kawachi T. Shiozuka M. Yamada S. Matsuda R. (2011). Mitochondrial adaptations in skeletal muscle to hindlimb unloading. Mol. Cell. Biochem. 350, 1–11. doi: 10.1007/s11010-010-0677-1, PMID: 21165677

[ref132] Wang L. Mascher H. Psilander N. Blomstrand E. Sahlin K. (2011). Resistance exercise enhances the molecular signaling of mitochondrial biogenesis induced by endurance exercise in human skeletal muscle. J. Appl. Physiol. 111, 1335–1344. doi: 10.1152/japplphysiol.00086.2011, PMID: 21836044

[ref133] Watson A. R. Dai H. Zheng Y. Nakano R. Giannou A. D. Menk A. V.. (2019). mTORC2 deficiency alters the metabolic profile of conventional dendritic cells. Front. Immunol. 10:1451. doi: 10.3389/fimmu.2019.01451, PMID: 31338091 PMC6626913

[ref134] Wen Y. Alimov A. P. Mccarthy J. J. (2016). Ribosome biogenesis is necessary for skeletal muscle hypertrophy. Exerc. Sport Sci. Rev. 44, 110–115. doi: 10.1249/JES.0000000000000082, PMID: 27135313 PMC4911282

[ref135] Wilkinson S. B. Phillips S. M. Atherton P. J. Patel R. Yarasheski K. E. Tarnopolsky M. A.. (2008). Differential effects of resistance and endurance exercise in the fed state on signalling molecule phosphorylation and protein synthesis in human muscle. J. Physiol. 586, 3701–3717. doi: 10.1113/jphysiol.2008.153916, PMID: 18556367 PMC2538832

[ref136] Wu Z. Puigserver P. Andersson U. Zhang C. Adelmant G. Mootha V.. (1999). Mechanisms controlling mitochondrial biogenesis and respiration through the thermogenic coactivator PGC-1. Cell 98, 115–124. doi: 10.1016/S0092-8674(00)80611-X, PMID: 10412986

[ref137] Yan W. Zhang H. Liu P. Wang H. Liu J. Gao C.. (2013). Impaired mitochondrial biogenesis due to dysfunctional adiponectin-AMPK-PGC-1alpha signaling contributing to increased vulnerability in diabetic heart. Basic Res. Cardiol. 108:329. doi: 10.1007/s00395-013-0329-1, PMID: 23460046

[ref138] Yeo W. K. Paton C. D. Garnham A. P. Burke L. M. Carey A. L. Hawley J. A. (2008). Skeletal muscle adaptation and performance responses to once a day versus twice every second day endurance training regimens. J. Appl. Physiol. 105, 1462–1470. doi: 10.1152/japplphysiol.90882.2008, PMID: 18772325

